# Relationship Between Smoking Habit and Sperm Parameters Among Patients Attending an Infertility Clinic

**DOI:** 10.3389/fphys.2019.01356

**Published:** 2019-10-31

**Authors:** Rehana Rehman, Nida Zahid, Sofia Amjad, Mukhtiar Baig, Zohair Jamil Gazzaz

**Affiliations:** ^1^Department of Biological and Biomedical Sciences, The Aga Khan University, Karachi, Pakistan; ^2^Department of Surgery, The Aga Khan University, Karachi, Pakistan; ^3^Department of Physiology, Ziauddin University, Karachi, Pakistan; ^4^Department of Clinical Biochemistry, Faculty of Medicine, King Abdulaziz University, Jeddah, Saudi Arabia; ^5^Department of Medicine, Faculty of Medicine, King Abdulaziz University, Jeddah, Saudi Arabia

**Keywords:** cortisol, FSH, dismutase, glutathione peroxidase, male infertility, oxidative stress, smoking, sperm motility

## Abstract

**Background:**

This study aimed to estimate stress markers, oxidative stress (OS), reproductive hormones and sperm parameters in male smokers and non-smokers and observe the impact of oxidative stress markers and smoking on sperm count, motility and morphology in a selected population of Karachi, Pakistan.

**Methods:**

This cross-sectional study was conducted from July 2017 to July 2018 at Aga Khan University (AKU), in Karachi, Pakistan. The subjects were recruited from the Sindh Institute of Reproductive Medicine (SIRM), Karachi based on defined inclusion criteria. The subjects were categorized into fertile and infertile based on cut off values of sperm parameters as recommended by the WHO i.e., sperm count/ejaculate of 39 × 10^6^/ml, sperm motility 40% and normal morphology 4%. Two hundred eleven fertile and 165 infertile male subjects were included in the study. Serum cortisol, adrenaline, superoxide dismutase (SOD), and glutathione peroxidase (GPX) were analyzed by ELISA kits. Data was analyzed on SPSS-22. A *p*-value of <0.05 was considered statistically significant.

**Results:**

Age, Body Mass Index (BMI), and body fat were similar among smokers and non-smokers. Age was significantly lower, while mean BMI and body fat were significantly higher among infertile smokers vs. fertile smokers (*p*-value < 0.05). The testosterone levels were significantly reduced among smokers as compared to non- smokers (*p*-value < 0.05). The median cortisol levels were increased as well as GPX, and steroid hormone-binding globulin (SHBG) were significantly reduced among smokers as compared to non-smokers. Additionally, the same findings with a significant difference have also been observed among infertile smokers as compared to fertile smokers (*p*-value < 0.05). This study has shown that the semen parameters (total count, motility, and morphology) are decreased in infertile smokers as compared to infertile non-smokers. Furthermore, the multivariate analysis showed that smoking causes a significant decrease in sperm count and morphology but it did not have any significant effect on motility.

**Conclusion:**

Smoking has a significant effect on fertility, specifically sperm count and normal morphology of sperm. This might be due to OS produced by smoking, which has devastating effects on semen parameters, thus reducing male fertility. Infertility specialist should counsel their patients about the ill effects of smoking on their fertility status and should advise maintaining a healthy lifestyle, including normal weight and avoiding smoking, to prevent future health problems. Hence smokers should quit smoking for their next generation.

## Introduction

Infertility is a rising epidemic, and male infertility is contributing equally. The preventable factors, the social costs of male infertility, and the resulting burden on public health need to be identified and overcome (Gaskins et al., 2014).

Smoking is amongst the modifiable risk factors of reproductive health and approximately 37% of men of reproductive age smoke cigarettes (Sharma et al., 2016). Consumption of tobacco is increasing globally, particularly in developing countries (Ng et al., 2014). Smoking causes six million deaths each year directly, and 600,000 deaths indirectly per year from second-hand smoke exposure (Low and Binns, 2013). Toxins from tobacco smoking have adverse effects on semen quality, including semen volume, sperm density, motility, viability, and normal morphology, thus causing male infertility (Dai et al., 2015). In addition to its link with male fertility impairment, tobacco smoking is also accountable for an upsurge in DNA injury, aneuploidies, and sperm mutations (Beal et al., 2017) and increased apoptosis of spermatogenic cells (Alsaed Laith and Barkhudarov, 2018).

Smoking has also been seen to cause OS in spermatozoa, due to formation of excessive reactive oxygen species (ROS) as a large amount of polyunsaturated fatty acids are present in the plasma membrane. In addition to this, decreased concentration of scavenging enzymes in the cytoplasm of spermatozoa causes the low antioxidant capacity (Harlev et al., 2015). Hence OS is imminent due to this excessive ROS and limited antioxidant defense mechanisms. Spermatozoa also are most susceptible to oxidative stress and oxidative DNA damage (ODD) due to their limited capacity for detection and repair of DNA damage (Bisht et al., 2017). Oxidative stress causes defective sperm function by affecting its viability, motility, DNA fragmentation, and membrane lipid peroxidation. One of the antioxidant, superoxide dismutase (SOD) has reported to be an important marker of male fertility (Murawski et al., 2007). This enzyme protects sperm from ODD, lipid peroxidation, and mitochondrial DNA impairment, decreased motility of the sperms (Wagner et al., 2018). Furthermore, another antioxidant enzyme, glutathione peroxidase (GPX) has also found to be essential for normal sperm function and its integrity due to its defensive action against ROS (Schneider et al., 2009; Wagner et al., 2018). However, the literature reported unpredictable and contradictory data regarding the impact of smoking on male infertility (Harlev et al., 2015).

Due to these inconsistencies in the literature about the effects of smoking on human fertility and furthermore lack of relevant published studies in this regard in our part of the world, pursued us to explore any link between smoking, stress markers, OS, reproductive hormones, and sperm parameters in infertile male subjects in our local setup. Hence, this study aimed to investigate the effect of oxidative stress, reproductive hormones and sperm parameters (sperm count, motility, and morphology) in infertile males with and without smoking in a selected population of Karachi, Pakistan.

## Subject and Method

The present cross-sectional study carried out from July 2017 to July 2018 at AKU, in Karachi, Pakistan. The subjects were recruited from the SIRM, Karachi, after approval by the Ethical Committee of AKU, Karachi, Pakistan (4813-BBS-ERC-17). The sample size was calculated on Open Epi software version 3.01. A sample size of 398 was estimated to compare the mean cortisol, GPX, SOD, and adrenaline levels in fertile and infertile men adjusting for 10% non-response rate. The sample size based on a level of significance of 5%, power for detecting the real effect of 80% and the anticipated difference in mean levels of the stress markers among fertile and infertile males of −0.27 (Shukla et al., 2010). However, final recruitment was of 376 males on account of many refusals to provide semen samples.

All male subjects with an age ranging from 25 to 45 years who approached the infertility center (irrespective of the cause of infertility) during the study period and who consented to take part in the study were included. According to the number of cigarettes smoked per day and duration of cigarettes smoked in a year, we categorized participants as either light smokers (1–10 cigarettes), moderate smokers (11–20 cigarettes) or heavy smokers (21–40 cigarettes) and short-term smokers (1–10 years) and long-term smokers (11–20 years).

### Semen Analysis

Participants provided semen samples by masturbation after abstinence of 2–3 days into a labeled sterile plastic specimen container. The specimen was collected either in the infertility clinic or was brought from home within an hour after the discharge. It was allowed to liquefy at 37°C for 20 min, and then normal saline was applied for sperm washing to remove the seminal plasma. The physical properties were assessed by macroscopic examination, which included appearance, volume, consistency, and pH. Improved Neubauer chamber was used for sperm count. A simple system of grading motility was used that distinguishes spermatozoa with progressive or non-progressive motility from those that are immotile.

Progressive motility (PR) was considered when spermatozoa were moving actively, either linearly or in a large circle and in non-progressive motility (NP) all other patterns of motility present with an absence of progression, e.g., swimming in small circles, the flagellar force hardly displacing the head, or when only a flagellar beat can be observed. Immotile (IM) had no movement.

Regarding sperm morphology, all spermatozoa with smooth rimmed oval shaped head, an acrosome tip covering about “40–70% of the sperm head”, mid-piece of the same length as head, and an uncoiled 45 μm long tail with no neck, head or tail defects were considered as normal. Semen samples showing <4% spermatozoa of normal morphology were considered as abnormal (WHO, 2010) (Cooper et al., 2010). One technician scored all slides to prevent inter-technician inconsistency in scoring and evaluation of the physical properties of semen was also done.

#### Division of Study Subjects

A physician thoroughly screened all the subjects for systemic or andrological pathologies. They were divided on the basis of (i) sperm parameters, and (ii) fertility history as is explained below.

##### Fertile group

The “fertility status” of study participants was based on the history that they have had kid with their wives in last 2–3 years and semen parameters analysis normozoospermic according to WHO criteria, i.e., “had total sperm number (TC) >39 million per ml, total sperm motility (progressive and non-progressive) measured within 60 min of collection of more than 40%, and normal morphology (MORPH) of ≥4%) (Cooper et al., 2010).

##### Infertile group

Men with a history of primary infertility with a sperm count of less than 15 × 10^6^, decreased total sperm motility of less than 40% and normal morphology of less than 4% were included. It comprised of all variants of azoospermia, oligozoospermia, teratozoospermia, and asthenozoospermia.

Subjects who had a female cause of infertility, suffered from secondary infertility and having cryptorchidism, testicular trauma, orchitis, and testicular hypotrophy (confirmed on history and examination) were excluded. Subjects having diabetes, hypertension, arthritis, malignancy, epilepsy, tuberculosis, endocrinal disorders, liver/renal disease (confirmed on laboratory investigations) and on drugs and conditions are known to influence OS and serum cortisol level were excluded. Moreover, subjects who were receiving testosterone or thyroxin replacement therapy or steroids were also excluded.

Two hundred and eleven fertile and 165 infertile male subjects [azoospermic 62 (37.57%), oligospermia 84 (50.90%), teratospermia 7 (4.24.%), asthenospermia 12 (7.27%)] were included in the study. The general physical examination, assessment of height, weight, and calculation of BMI was done, and estimation of body fat% age was performed by a Bioelectrical Impedance Analyzer (BIA). Gender, age, and height were manually entered into the system by a digital keyboard, and percentage of fat mass (% FM) was calculated.

Andrologic profile of fertile group was done to compare any significant difference between fertile and infertile group and to further confirming that there is no andrologic imbalance present between the groups.

Blood samples were collected from the subjects at 8 AM, and the serum was separated, centrifuged, and analyzed. Serum FSH was determined by using the commercially available kits for Human FSH Enzyme Immunoassay (Kit Cat. No KAPD1288 by DIA source Immuno Assays S.A. Belgium) and detection range for FSH for male was 1–14 mIU/ml. Serum LH estimation was made by using the commercially available kits for Human LH Enzyme Immunoassay (Kit Cat. No KAPD1289 by DIA source Immuno Assays S.A. Belgium) and detection range for LH for male was 0.7–7.4 mIU/ml. Serum total testosterone was determined by using the commercially available kits for Human Total Testosterone (TT) Enzyme Immunoassay (Kit Cat. No DKO002 by DIA source Immuno Assays S.A. Belgium) and detection range for TT for adult male was 2.12 – 6.01 ng/mL. Cortisol was analyzed using the Cortisol ELISA kits (catalog number: CO103S, CALBIOTECH) with a detection range of 50–230 ng/mL from 8:00 am -11:00 am and 30–150 ng/mL at 4:00 pm. Adrenaline was analyzed using Human Epinephrine/Adrenaline (EPI) ELISA kits (catalog number: 95362, Glory Science Co., Ltd.), a detection range of 5 ng/L-1000 ng/L. SOD was analyzed using Human SOD ELISA kits (catalog #: 11086, Glory Science Co., Ltd.) according to the manufacturer protocol and the detection range for SOD kit was 3 – 160 mU/L. GPX was analyzed using Human GPX ELISA kits (catalog #: 10800, Gl Science Co., Ltd.), which has a detection range of 30–700 U/L. SHBG was analyzed using kits (catalog #: 10222, Glory Science Co., Ltd.) and detection range for SHBG kit was 2–40 nmol/l, which had an intra-assay and intraassay coefficient of 9 and 15%, respectively.

Data analysis was performed using Statistical Package for the Social Sciences (SPSS), version 22.0, and STATA version 12. Descriptive statistics were computed for categorical variables by computing their frequencies and percentages, and Chi-square/Fisher’s exact test was used to calculate the difference. The distribution of quantitative variables was computed by their means and standard error/median (IQR) depending on the normality of the data and assessed by independent *t*-test/Mann Whitney test. Unadjusted and adjusted beta coefficients with their 95% CI were reported by using simple and multiple linear regression. For univariate analyses, the cutoff used for identifying variables for the multivariable model was *p*-value of less than 0.25. The purpose of using a significance level as high as 0.25 as a screening criterion for initial variable selection is based on the work by Bendel and Afifi (1977) on linear regression (Hosmer et al., 2013). The authors showed that use of a more traditional level (such as 0.05) often fails to identify variables known to be important. Use of the higher level has the disadvantage of including variables that are of questionable importance at this initial stage of model development. For this reason, it is important to review all variables added to a model critically before a decision is reached regarding the final model, therefore, for multivariable analysis, the *p*-value of <0.05 was used.

## Results

### Demographics, Sex Hormones, Stress Makers, and Semen Parameters Among Smoker and Non-smokers

The study population consisted of 376 male participants. Smoking status was classified as follows: 272 (72%) were smokers, and 104 (28%)were non-smokers, 40%(109/272) were short-term smokers, and 60% (163/272) were long-term smokers. There were 36% (99/272) light smokers, 44% (119/272) moderate smokers, and 20% (54/272) who were heavy smokers.

Mean age, BMI, and body fat were similar among smokers and non-smokers. The mean testosterone levels were significantly lower among smokers as compared to non-smokers (*p*-value = 0.02). However, there was no significant difference in FSH and LH levels between the two groups. A significant difference was observed in the levels of stress markers (adrenaline, SOD, and GPX) between smokers and non-smokers. Significant lower levels of adrenaline, SOD, glutathione reductase (*p*-value < 0.001) and SBHG (*p*-value = 0.03) were evident among smokers as compared to non-smokers. However, cortisol levels were decreased in non-smokers as compared to the smokers’ group. No difference was found in semen parameters between the groups (Table 1).

**TABLE 1:** Demographics, sex hormones, stress makers and semen parameters among smoker and non-smokers.

**Demographics/sex hormones/stress markers/semen parameters**	**Smokers (*n* = 272)**	**Non-smokers (*n* = 104)**	***p*-value**
**Demographics**			
Age (Years) Mean ± SE	35.09 ± 0.37	35.67 ± 0.63	0.42
Range	33	37	
BMI (Kg/m^2^) (Mean ± SE)	26.01 ± 0.22	26.14 ± 0.30	0.74
Range	19.03	14.47	
BMI (Kg/m^2^)			0.59
Normal weight 18–22.9	60 (22.10%)	18 (17.30%)	
Over weight 23–24.9	44 (16.25%)	18 (17.30%)	
Obese ≥25	168 (61.80%)	68 (65.40%)	
Body fat (%) (Mean ± SE)	33.88 ± 0.28	34.17 ± 0.39	0.57
Body fat (%)			0.51
Normal weight (12–22)	0 (0.0%)	4 (1.50%)	
Over weight (22.1–27)	7 (6.70%)	14 (5.10%)	
Obese (>27)	97 (93.30%)	254 (93.45)	
**Hormones**			
FSH (in mIU/ml) Mean ± SE	9.48 ± 0.08	9.55 ± 0.13	0.67
Range	7.70	6.24	
LH (in mIU/ml)Mean ± SE	9.92 ± 0.22	9.26 ± 0.35	0.12
Range	15.9	4.08	
Testosterone (in ng/ml), Mean ± SE	4.31 ± 0.11	4.78 ± 0.16	0.02^∗^
Range	8.88	8.33	
SHBG Median (in nmol/L) (IQR)	18.95 (15.04–28.82)	22.53 (16.49–30.71)	0.03^∗∗^
**Stress markers**			
Cortisol (in ug/ml) Median (IQR)	139.21(109.22–181.69)	123.74(81.17–209.04)	0.15
Adrenaline(in ng/L) Median (IQR)	24.78 (14.40 - 41.42)	47.05 (22.53–60.92)	<0.001^∗∗^
**Semen parameters**			
Total count (ml) Mean ± SE	77.34 ± 3.420	86.07 ± 5.24	0.17
Motility (%) Median (IQR)	65.00 (42.75–75.00)	70.00 (45.00- 75.00)	0.17
Morphology (%) Mean ± SE	4.75 ± 0.23	5.65 ± 0.42	0.05

*^∗^Significant at *p*-value < 0.05 by using independent *t*-test. ^∗∗^Significant at *p*-value < 0.05 by using Mann Whitney *U* test.*

### Comparison of Demographics, Sex Hormones, and Stress Makers Among Infertile and Fertile Smokers

The Table 2A shows that age was significantly lower while mean BMI and body fat were significantly higher among infertile smokers vs. fertile smokers (*p*-value < 0.05). A higher mean testosterone level was observed among fertile smokers as compared to infertile smokers (*p*-value < 0.05). Among stress markers, the median cortisol and SOD were higher among infertile smokers as compared to fertile smokers (*p*-value < 0.05). On the contrary, the median GPX and SHBG were significantly lower among infertile smokers as compared to fertile smokers.

**TABLE 2:** Comparison of Demographics, sex hormones and stress makers among infertile and fertile smokers/fertile and infertile non-smokers.

**Demographics/sex hormones/stress markers/semen parameters**	**(A) Smokers (*n* = 272)**		**(B) Non-smokers (*n* = 104)**	
		
	**Infertile (125)**	**Fertile (147)**	**Infertile (40)**	**Fertile (64)**
**Demographics**				
Age (Years)^∗^^#^ Mean ± SE	34.29 ± 0.52	35.77 ± 0.53	33.20 ± 0.94	37.22 ± 0.78
Range	27	31	25	32
BMI (Kg/m^2^) ^∗^ (Mean ± SE)	26.93 ± 0.33	25.22 ± 0.28	26.03 ± 0.55	26.21 ± 0.36
Range	18.26	14.15	14.47	12.04
BMI (Kg/m^2^)^∗^^				
Normal weight 18–22.9	20 (16.00%)	40 (27.20%)	9 (22.50%)	9 (14.10%)
Over weight 23–24.9	18 (14.40%)	26 (17.70%)	6 (15.00%)	12 (18.80%)
Obese ≥25	87(69.60%)	81 (55.10%)	25 (62.50%)	43 (67.20%)
Body fat (%)^∗^ (Mean ± SE)	34.81 ± 0.43	33.09 ± 0.36	33.47 ± 0.66	34.62 ± 0.48
Body fat (%)				
Normal weight (12–22)	1 (0.80%)	3 (2.00%)	0 (0.00%)	0 (0.00%)
Over weight (22.1–27)	4 (3.20%)	10 (6.80%)	4 (10.00%)	3 (4.70%)
Obese (>27)	120 (96.00%)	134 (91.20%)	36 (90.00%)	61 (95.30%)
**Hormones**				
FSH (in mIU/ml) Mean ± SE	9.48 ± 0.12	9.49 ± 0.11	9.49 ± 0.21	9.58 ± 0.17
Range	6.21	7.7	4.51	6.24
LH (in mIU/ml) Mean ± SE	10.19 ± 0.32	9.69 ± 0.30	9.39 ± 0.63	9.19 ± 0.42
Range	14.86	14.8	13.73	12.48
Testosterone (in ng/ml)^∗^, Mean ± SE	3.97 ± 0.16	4.60 ± 0.14	4.63 ± 0.25	4.87 ± 0.21
Range	8.88	8.88	7.13	8.03
SHBG (in nmol/L) ^∗∗^ Median (IQR)	18.14 (14.22–21.79)	21.93 (16.12–33.82)	21.79 (17.66–26.79)	22.94 (15.44–31.89)
**Stress Markers**				
Cortisol (in ug/ml) ^∗∗^ Median (IQR)	155.35(115.03–210.81)	127.41 (94.43–168.16)	100.94 (79.85–200.94)	127.41 (83.94–217.98)
Adrenaline (in ng/L) Median (IQR)	24.19 (13.45–42.38)	25.42 (14.41–40.74)	49.19 (22.84–62.80)	47.06 (22.32–60.57)
SOD(in ng/ml)^∗∗^ Median (IQR)	16.03 (8.08–27.63)	11.35 (5.90–27.63)	32.73 (18.66-67.95)	43.92 (18.66–134.89)
Glutathione(in pg/ml)^∗∗^ Median (IQR)	48.44 (37.79–78.82)	60.66 (42.60–87.58)	345.87 (93.68–618.42)	288.82(129.05–618.42)

*^∗^Significant at *p*-value < 0.05 using independent *t*-test to assess the relationship of normally distributed quantitative variables with fertility status among smokers ^#^Significant at *p*-value < 0.05 using independent *t*-test to assess the relationship of normally distributed quantitative variables with fertility status among non-smokers. ^∗^^Significant at *p*-value < 0.05 by using chi-square test to assess the relationship of qualitative variable with fertility status among smokers. ^∗∗^Significant at *p*-value < 0.05 using Mann whitney *U* test to assess the relationship of non-normal quantitative variables with fertility status among smokers.*

### Comparison of Demographics, Sex Hormones and Stress Makers Among Fertile and Infertile Non-smokers

The Table 2B shows that the mean age, BMI, and body fat were not significantly different among the infertile non-smokers and fertile non-smokers. No substantial changes were observed in the level of stress markers (cortisol, adrenaline, SOD, glutathione, and SBHG) between infertile non-smokers and fertile non-smokers.

### Univariate and Multivariate Analysis for Assessing the Relationship of Smoking and Stress Markers With Sperm Parameters

#### Total Sperm Count

The relationship of the stress markers; cortisol, adrenaline, SOD, and glutathione were also assessed with the total sperm count and a significant decrease was found in total sperm count with every one-unit increase in cortisol and adrenaline levels. However, there was a significant increase in total sperm count with an increase in SHBG levels. There was no significant relationship observed between total sperm count with SOD and glutathione at *p*-value < 0.25 (Table 3).

**TABLE 3:** Univariate and Multivariate analysis for assessing the relationship of smoking and stress markers with total sperm count among male patients.

**Demographics/sex hormones/stress markers**	**Univariate analysis**		**Multivariate analysis**	
		
	**ß (SE)**	**95% CI**	**ß (SE)**	**95% CI**
**Smoking status**				
Non-smoker (ref)	–			
Smoker	−8.72(6.41)	−21.33, 3.88^∗^	−17.76(8.03)	−33.56, −1.96^∗∗^
Cortisol	−0.11(0.04)	−0.19, −0.03^∗^	−0.08 (0.04)	−0.16, −0.004
Adrenaline	−0.07(0.057)	−0.19, 0.03^∗^	−0.07 (0.05)	−0.18, 0.04
SOD	0.05(0.05)	−0.05, 0.15	0.16 (0.10)	−0.03, 0.36
Glutathione	0.006(0.01)	−0.01, 0.030	−0.05 (0.02)	−0.10, 0.0001^∗∗^
BMI (Kg/m^2^)	−3.50(0.79)	−5.06, −1.95^∗^	−3.41 (0.79)	−4.98, −1.84^∗∗^
BMI (Kg/m^2^)				
Normal weight 18−22.9 (ref)	–		NS	
Over weight 23−24.9	−20.20(9.29)	−38.47, −1.93^∗^		
Obese ≥25	−29.377(7.132)	−43.40, 15.35^∗^		
Age (Years)	0.74(0.45)	−0.148, 1.64^∗^	NS	
Body fat (%)	−2.254(0.62)	−3.485, −1.02^∗^	NS	
Body fat (%)				
Normal weight (12−22) (ref)	15.27(30.41)	−75.079, 44.53		
Over weight (22.1−27)	23.71(28.03)	−78.843, 31.40		
Obese (>27)				
FSH (mIU/ml)	0.83(2.11)	−3.54, 6.54^∗^	NS	
LH (mIU/ml)	−2.01(0.78)	−3.54, −0.47^∗^	NS	
Testosterone (ng/dl)	3.44(1.57)	0.346, 6.55^∗^	NS	
SHBG	0.43(0.18)	0.076, 0.78^∗^	NS	

*^∗^Significant at *p*-value < 0.25 by univariate linear regression. ^∗∗^Significant at *p*-value < 0.05 by multivariate linear regression.*

Multivariate analysis showed that after adjusting for the covariates among smokers, the total sperm count was 17.7 million lower as compared to non-smokers. Moreover, the total sperm count was significantly decreased with one-unit increase in cortisol levels, glutathione levels and BMI at *p*-value < 0.05. However, no significant relationship was observed between adrenaline and SOD with total sperm count at *p*-value < 0.05 (Table 3).

#### Sperm Motility

In univariate analysis, it was observed that the sperm motility was increased with every one-unit increase in age, testosterone levels, SOD, and glutathione. However, sperm motility was decreased with 1 every unit increase in BMI,% body fat, LH, cortisol, adrenaline, and SHBG No significant relationship was observed between smoking status and FSH with sperm motility at *p*-value < 0.25 (Table 4).

**TABLE 4:** Univariate analysis and Multivariate analysis for assessing the relationship of smoking and stress markers with sperm motility among male patients.

**Demographics/sex hormones/stress markers**	**Univariate analysis**		**Multivariate analysis**	
		
	**ß (SE)**	**95% CI**	**ß (SE)**	**95% CI**
**Smoking status**				
Non-smoker (ref)	–			
Smoker	−3.177(2.83)	−8.75, 2.39	−5.251 (3.37)	−11.88, 1.38
Cortisol	−0.09(0.01)	−0.12, −0.058^∗^	−0.06 (0.01)	−0.10, −0.03^∗∗^
Adrenaline	−0.06(0.025)	−0.11, 0.01^∗^	−0.061 (0.024)	−0.10, 0.012^∗∗^
SOD	0.04(0.02)	0.002, 0.09^∗^	0.09(0.04)	−0.01, 0.17^∗∗^
Glutathione	0.01(0.005)	−0.001, 0.01^∗^	−0.02 (0.01)	−0.04, 0.0008
Age (Years)	0.654(0.19)	0.26, 1.04^∗^	0.62 (0.18)	0.23, 0.99^∗∗^
BMI (Kg/m^2^)	−1.88(0.34)	−2.56, −1.20	−1.72 (0.33)	−2.38, −1.06^∗∗^
BMI (Kg/m^2^)				
Normal weight 18−22.9 (ref)	–			
Over weight 23−24.9	−0.73(4.10)	−8.79, 7.33^∗^		
Obese ≥25	−10.99(3.14)	−17.18, −4.80	NS	
Body fat (%)	−1.10(0.27)	−1.64, −0.55^∗^		
Body fat (%)			NS	
Normal weight (12−22) (ref)	−			
Over weight (22.1−27)	−6.52(13.38)	−32.84,19.79		
Obese (>27)	−14.98(12.33)	−39.24, 9.27		
FSH	0.79(0.93)	−1.03, 2.62	NS	
LH	−1.006(0.34)	−1.68, −0.32^∗^	NS	
Testosterone	2.56(0.68)	1.21, 3.92^∗^	NS	
SHBG	0.36(0.07)	0.21, 0.51^∗^	NS	
Cortisol	−0.09(0.01)	−0.12, 0.058^∗^	NS	
Adrenaline	−0.06(0.02)	−0.11, 0.01^∗^	NS	
SOD	0.04(0.02)	0.002, 0.09	NS	
Glutathione	0.008(0.005)	−0.001, 0.01^∗^	NS	

*^∗^Significant at *p*-value < 0.25 by univariate linear regression. ^∗∗^Significant at *p*-value < 0.05 by multivariate linear regression.*

In the multivariate analysis, it was observed that the sperm motility decreased 5.25% among smokers as compared to non-smokers. Moreover, with every one-unit increase in cortisol, adrenaline, and BMI the sperm motility was significantly decreased, while with every one-unit increase in SOD and age, the sperm motility was significantly increased (Table 4).

#### Sperm Morphology

In univariate analysis, it was observed that with every one-unit increase in age, testosterone, SHBG, SOD, and glutathione, the expected normal sperm morphology was significantly increased, while, with every 1 unit increase in BMI,% body fat, LH and cortisol the normal sperm morphology was significantly decreased. Among smokers, the normal sperm morphology was 0.89% lower as compared to non-smokers (Table 5).

**TABLE 5:** Univariate and multivariate analysis for assessing the relationship of smoking and stress markers with sperm morphology among male patients.

**Demographics/sex hormones/stress markers**	**Univariate analysis**		**Multivariate analysis**	
		
	**ß (SE)**	**95% CI**	**ß (SE)**	**95% CI**
**Smoking status**				
Non-smoker (ref)	–			
Smoker	−0.89(0.46)	−1.80,0.014^∗^	−1.29 (0.57)	−2.42, −0.17^∗∗^
Cortisol	−0.01(0.002)	−0.01, −0.006^∗^	−0.008 (0.003)	−0.01, −0.002^∗∗^
Adrenaline	−0.002(0.004)	−0.01,0.006	−0.002 (0.004)	−0.01, 0.005
SOD	0.009(0.003)	0.002,0.016^∗^	0.015 (0.007)	0.001, 0.02^∗∗^
Glutathione	0.001(0.001)	−0.0001,0.003^∗^	−0.003 (0.001)	−0.007, −0.0001^∗∗^
SHBG	0.06(0.01)	0.03, 0.08^∗^	0.038 (0.01)	0.01, 0.06^∗∗^
BMI (in kg/m^2^)	−0.21(0.05)	−0.32, −0.10^∗^	−0.14(0.05)	−0.26, −0.03^∗∗^
BMI (in kg/m^2^)				
Normal weight 18−22.9 (ref)	–			
Over weight 23−24.9	−0.09(0.68)	−1.43,1.24^∗^		
Obese≥25	−1.19(0.52)	−2.22, −0.16		
Age (in years)	0.04(0.03)	−0.01,0.11^∗^	NS	
Body fat (in %)	−0.13(0.04)	−0.22, −0.047^∗^	NS	
Body fat (in %)				
Normal weight (12−22) (ref)	–			
Over weight (22.1–27)	−1.54, 2.19	−5.86,2.77^∗^		
Obese (>27)	−2.57, 2.02	−6.56, 1.40		
FSH	−0.0.24(0.15)	−0.32,0.27	NS	
LH	−0.17(0.05)	−0.28, −0.06^∗^	NS	
Testosterone	0.59(0.11)	0.375,0.81^∗^	NS	

*^∗^Significant at *p*-value < 0.25 by univariate linear regression. ^∗∗^Significant at *p*-value < 0.05 by multivariate linear regression.*

In multivariate analysis, the normal sperm morphology was 1.29% lower among smokers as compared to non-smokers. Moreover, with every one-unit increase in BMI, cortisol, and glutathione, the normal sperm morphology was significantly decreased. Besides, with every one-unit increase in SOD and SHBG, the normal sperm morphology was significantly increased (Table 5).

## Discussion

Smoking is an eminent risk factor for reproductive health (Kumar et al., 2014). Smoking causes reproductive hormone disorders, impairment in spermatogenesis and maturation of sperm, and compromised functioning of spermatozoa (Saleh et al., 2002). This study has shown a significant decrease in testosterone level in smokers as compared to non-smokers. Many tobacco smoke components have effects on testosterone. Nicotine has been reported to impair male reproductive hormone system by causing Leydig cell apoptosis and inhibition of synthesis of androgen (Dai et al., 2015). Toxins, including lead present in tobacco, appear to directly impair the process of spermatogenesis itself as well as sperm function through reproductive axis dysfunction or testicular degeneration (Gandhi et al., 2017). In contrast to the present study, a recent study reported no impact of smoking on total testosterone and SHBG (Boeri et al., 2019).

This study has shown that the semen parameters (total count, motility, and morphology) are decreased in infertile smokers as compared to infertile non-smokers. Smoking caused a significant decrease in sperm count and morphology. However, it did not have any significant effect on motility in this study subjects. These results are similar to a few other studies (Sharma et al., 2016; Mostafa et al., 2018). A recent study has also found a significantly higher incidence of morphological defects of spermatozoa among smokers and no difference in motility and reproductive hormones in both groups (Bundhun et al., 2019).

The current study observed significantly lower levels of GPX and SHBG in smokers. Among stress markers, the median cortisol levels were significantly higher among smokers as compared to non-smokers. Increased cortisol levels have an inhibitory effect on male reproductive function that could be due to a significant increase in intracellular levels of ROS and induction of apoptosis of Leydig cells (Darbandi et al., 2018). Smoking is one of the contributing factors to OS, produced due to excessive levels of ROS coupled with a deficiency in antioxidants. Though low levels of ROS are required for sperm capacitation, hyperactivation, acrosome reaction, and spermatozoa–oocyte fusion but their excess overwhelms the neutralizing capacity of antioxidants thus causes OS, and consequent damage to DNA in the nucleus and mitochondria (Bui et al., 2018). This alteration in the sperm quality due to oxidative stress-induced DNA damage is due to the active transfer of cigarette components through the blood-testis barrier (Sepaniak et al., 2004). Tobacco contains mutagenic and carcinogenic substances (i.e., nicotine, cadmium, and lead), which are damaging to cells undergoing rapid multiplication, including germ cells in the testis (Fariello et al., 2012) Hence, oxidative stress affects fertility by affecting sperm viability, motility, DNA fragmentation, and membrane lipid peroxidation (Gandhi et al., 2017). Literature indicates that the molecular changes produced by OS are nuclear and mitochondrial DNA damage, telomere shortening, epigenetic alterations, and Y chromosomal micro-deletions, which leads to male infertility (Bui et al., 2018). Moreover, smoking-induced increase in inflammatory agents affects sperm genome, gonads and sperm-ovum fecundation leading to infertility (Davar et al., 2012) and cigarette smoking also causes alterations in DNA methylation as well (Dong et al., 2017). This DNA damage leads to a higher risk of abortions, birth defects, and childhood cancers in the offspring (Gandhi et al., 2017). The adverse effect of tobacco on the paternal genome affects germ cell integrity and birth of healthy offspring because biological parenting begins much before the birth of a child and even before conception (Kumar et al., 2015).

The present study has shown that obesity has a significant negative effect on semen parameters. The effect of obesity on semen quality may be mainly due to disturbed endocrine mechanisms as there is a need for the controlled testicular environment and hypothalamic-pituitary-testicular axis for spermatogenesis (Agarwal et al., 2014). This may be due to the accumulation of toxic substances in the adipose tissue and endocrine disruptors (Oliveira et al., 2018). The accumulation of fatty tissue around the scrotum in obese men causes an increase in scrotal temperature and leads to oxidative stress in the testicles affecting the semen parameters (Oliveira et al., 2018).

This study has shown that an increase in age increases fertility, though some studies have been demonstrated that increasing paternal age is associated with decreased fertility rate (Shrem et al., 2019). Contrasting results of this study might be due to the fact that males aged 40–49 years found to be a 52% decrease in normal sperm motility, while it was markedly reduced by 79% at 50 years or older (Mariappen et al., 2018). In addition to this decline in sperm concentration has also not been observed with increasing male age (Johnson et al., 2015), that is why both the increase in motility and morphology have been found in this study.

Darbandi et al. (2018) proposed that normal functions of male reproductive hormones depend upon the balance between the ROS generation and antioxidant defense method in the reproductive system. Excessive production of ROS can affect reproductive tissues directly or may impede with the HPG axis and its relationship with other endocrine glands, thus causing infertility (Darbandi et al., 2018).

The present study did not categorize infertile patients according to sperm nomenclatures as per WHO criteria i.e., normozoospermic, teratozoospermia, oligozoospermia, (oligoteratozoospermia oligoasthenoteratozoospermia) and azoospermia. The insignificant difference in levels of FSH between the groups may be due to the greater number of obstructive azoospermic subjects who tend to have normal serum concentrations of FSH, LH, and testosterone (Jarvi et al., 2010; Jahan et al., 2011).

Thus it appears that smoking affects male fertility by affecting testosterone secretion, which could be due to OS potentiated by the smoking leading to decrease in semen parameters (total count and morphology). Therefore, it is suggested that all infertile couples who consult to fertility centers should be advised regarding lifestyle modifications, especially cessation of smoking and reduction of body weight. Such modifications would not only help to conceive but also improve the quality of life and prevent the other adverse impact of smoking and obesity in their life. Durairajanayagam (2018) recommended few lifestyle factors modifications like cigarette smoking, use of alcohol and illicit drugs, psychological stress, obesity, food, and caffeine consumption to avoid their detrimental effect on male fertility and to bring improvement in the fertility outcome (Durairajanayagam, 2018).

The main strength of the present study is that it evaluated the effect of smoking on semen parameters and stress markers, OS, reproductive hormones in a selected population of Karachi in Pakistan.

### Limitations

The present study represents the results of the heterogeneous group of infertile males, which could affect the characteristics of these subjects. New thematic research studies are needed to explore factors responsible for variation in sperm parameters leading to different attributes in infertile groups. Furthermore, this study could not assess participants’ anxiety, stress, and depression levels. Since it was a descriptive study, therefore, a strong causal connection could not be established.

## Conclusion

Smoking has a significant effect on fertility, specifically sperm count and normal morphology of sperm. This effect might be due to oxidative stress produced by smoking, which has devastating effects on semen parameters, thus reducing male fertility. Infertility specialist should counsel their patients about the ill effects of smoking on their fertility status and should advise maintaining a healthy lifestyle, including normal weight and avoiding smoking, to prevent future health problems. Hence smokers should quit smoking for their next generation.

## Data Availability Statement

The datasets generated for this study are available on request to the corresponding author.

## Ethics Statement

The approval for this study was granted by the Ethical Committee of AKU, Karachi, Pakistan (4813-BBS-ERC-17). Informed written consent was obtained from all participants of the study.

## Author Contributions

RR conceived, designed the study, arranged the experimental work, and contributed in manuscript writing. SA executed the study and helped in drafting the manuscript. NZ took part in data analysis, write up of results, and formulation of tables. MB and ZG supervised the project, and contributed in drafting the manuscript and arrangement for the experimental work. All authors read and approved the final version of the manuscript, and agreed with the order of presentation of the authors.

## Conflict of Interest

The authors declare that the research was conducted in the absence of any commercial or financial relationships that could be construed as a potential conflict of interest.
